# Accurate prediction of HCC risk after SVR in patients with hepatitis C cirrhosis based on longitudinal data

**DOI:** 10.1186/s12885-023-11628-1

**Published:** 2023-11-25

**Authors:** Yanzheng Zou, Ming Yue, Linna Jia, Yifan Wang, Hongbo Chen, Amei Zhang, Xueshan Xia, Wei Liu, Rongbin Yu, Sheng Yang, Peng Huang

**Affiliations:** 1 Department of Epidemiology, Center for Global Health, School of Public Health, Nanjing Medical University, Nanjing, 211166 China; 2 Department of Infectious Diseases, The First Affiliated Hospital of Nanjing Medical University, Nanjing, China; 3 Department of Infectious Disease, Jurong Hospital Affiliated to Jiangsu University, Jurong, China; 4 Faculty of Life Science and Technology, Kunming University of Science and Technology, Yunnan, China; 5 Kunming Medical University, Kunming, China; 6 Beijing Institute of Microbiology and Epidemiology, State Key Laboratory of Pathogen and Biosecurity, Beijing, China; 7 Department of Biostatistics, Center for Global Health, School of Public Health, Nanjing Medical University, Nanjing, 211166 China

**Keywords:** Hepatocellular carcinoma, Direct-acting antivirals, Machine learning, Longitudinal study, Predictive models

## Abstract

**Background:**

Most existing predictive models of hepatocellular carcinoma (HCC) risk after sustained virologic response (SVR) are built on data collected at baseline and therefore have limited accuracy. The current study aimed to construct an accurate predictive model incorporating longitudinal data using a novel modeling strategy. The predictive performance of the longitudinal model was also compared with a baseline model.

**Methods:**

A total of 400 patients with HCV-related cirrhosis who achieved SVR with direct-acting antivirals (DAA) were enrolled in the study. Patients were randomly divided into a training set (70%) and a validation set (30%). Informative features were extracted from the longitudinal variables and then put into the random survival forest (RSF) to develop the longitudinal model. A baseline model including the same variables was built for comparison.

**Results:**

During a median follow-up time of approximately 5 years, 25 patients (8.9%) in the training set and 11 patients (9.2%) in the validation set developed HCC. The areas under the receiver-operating characteristics curves (AUROC) for the longitudinal model were 0.9507 (0.8838–0.9997), 0.8767 (0.6972,0.9918), and 0.8307 (0.6941,0.9993) for 1-, 2- and 3-year risk prediction, respectively. The brier scores of the longitudinal model were also relatively low for the 1-, 2- and 3-year risk prediction (0.0283, 0.0561, and 0.0501, respectively). In contrast, the baseline model only achieved mediocre AUROCs of around 0.6 (0.6113, 0.6213, and 0.6480, respectively).

**Conclusions:**

Our longitudinal model yielded accurate predictions of HCC risk in patients with HCV-relate cirrhosis, outperforming the baseline model. Our model can provide patients with valuable prognosis information and guide the intensity of surveillance in clinical practice.

**Supplementary Information:**

The online version contains supplementary material available at 10.1186/s12885-023-11628-1.

## Background

Hepatitis C virus (HCV) infection remains a severe public health problem today, with an estimated 71 million chronically infected worldwide [1]. One of the most serious outcomes of HCV infection is the occurrence of hepatocellular carcinoma (HCC). HCC resulted in approximately 830,000 deaths in 2020 alone [2]. The new direct-acting antivirals (DAA) regime offers unprecedented rates of HCV virus eradication, with a sustained virologic response (SVR) rate of over 90% [3, 4]. With the widespread use of DAA, it is expected that most HCV-infected patients will achieve SVR after their antiviral treatments.

However, several studies have shown that the residual risk of HCC persists after HCV eradication [5–7]. Additionally, the risk of HCC increases in patients with cirrhosis [5]. Results of a large-scale cohort study revealed that patients with HCV-related cirrhosis presented a significantly higher annual incidence rate of HCC after SVR, well above the threshold of surveillance recommendation by the American Association for the Study of Liver Diseases [8]. Several factors, including older age [6], male gender [9], alpha-fetoprotein (AFP) [10, 11], aspartate aminotransferase (AST) [12], alanine transaminase (ALT) [12], gamma-glutamyl transferase (GGT) [13] and total bilirubin [13], were reported to be associated with the increased risk of HCC in patients with HCV or cirrhosis. Risk prediction models were constructed on these predictor variables to guide clinical decisions regarding the intensity of surveillance for cirrhotic patients who have reached SVR.

Most of the published predictive models were built on a few variables collected at baseline using conventional modeling strategies [11, 13, 14]. These models are usually mediocre in predictive performance since the risk of HCC can fluctuate over time as patients age, portal hypertension worsens, or liver stiffness increases [15]. In contrast, longitudinal models incorporating the repeated measurements of the predictor variables are able to capture the dynamic risk of HCC occurrence post-SVR. Furthermore, the longitudinal models can distinguish between patients who have similar values of predictor variables at baseline but continue to have different outcomes.

Machine learning algorithms have long been used in predictive modeling [16, 17]. The random survival forest (RSF) algorithm is regarded as a better alternative to the conventional Cox model in survival analysis [18]. Recently, a novel modeling framework has been developed that is capable of including information extracted from longitudinal data into RSF [19]. The current study aimed to construct a longitudinal predictive model using this modeling approach to predict HCC occurrence in patients with HCV-related cirrhosis. We also compared the performance of our longitudinal model with a baseline model.

## Methods

### Study population and follow-up

A total of 1042 patients with chronic hepatitis C from the Chronic Hepatitis C Research Program of Jiangsu (CHCRPJ) underwent DAA treatment from July 2012 to October 2020 at Jurong people’s hospital, China. Among these patients, 485 had been diagnosed with cirrhosis prior to treatment. Cirrhosis was diagnosed based either on a liver biopsy showing Metavir F4, a transient elastography score > 14 kPa, or clinical evidence. Patients who did not reach SVR after treatment, patients diagnosed with HCC prior to treatment, and patients who lacked the required serum biomarker values at baseline were further excluded. SVR was determined as a serum HCV RNA viral load below the lower limit of detection at least 12 weeks after completion of treatment. Eventually, 400 patients were enrolled in the study. The flow diagram of patient selection is presented in Figure S1.

The index date of the study was the start of DAA treatment. Patients were followed until HCC development, death or 31/11/2022, whichever came first. The study outcome was HCC occurrence after the index date. HCC was diagnosed according to the guidelines of the American Association for the Study of Liver Diseases [20]. Information on HCC occurrence both before and after treatment was retrieved from hospital inpatient and outpatient diagnoses. Patients not developing HCC were censored at the end of follow-up or the date of death.

Written informed consent was obtained from all participants for the use of their data. The study protocol complied with the ethical guidelines of the Declarations of Helsinki and Istanbul. The study was approved by the institutional ethics review committee of Nanjing Medical University.

### Predictor variables

The predictor variables involved in model development were selected based on their availability in the current study and their association with HCC described in previous literature. The predictor variables were classified into two categories, baseline predictors and longitudinal predictors. The baseline predictors, including age and gender, were collected at enrollment and did not change over time. The longitudinal predictors might change over time, as they were collected at enrollment and measured multiple times afterwards when patients returned for medical visits during the follow-up period. The longitudinal predictors were serum biomarkers, including AFP, total bilirubin, direct bilirubin, ALT, AST, cholinesterase, alkaline phosphatase (ALP), GGT, total protein, and albumin.

Patients attended follow-up visits at variable time intervals. At each visit, their serum biomarkers, including the aforementioned longitudinal predictors, were measured. If any measurement from a follow-up visit was missing one of the longitudinal predictors, the entire data from that visit was excluded. Consequently, the time intervals between the repeated measurements for each patient were irregular.

### Model development

We developed two types of models to predict HCC occurrence in patients reaching SVR–the longitudinal model and the baseline model for comparison. The longitudinal model was constructed in two steps following the modeling framework proposed by Lin et al [19]. First, to retrieve information from every repeatedly measured longitudinal variable, we used the fast covariance estimation method (FACEs) developed by Xiao et al [21]. FACEs is a new covariance-based functional principal component analysis (FPCA) method that has considerably expanded the applicability of functional data analysis to irregularly spaced data such as longitudinal data. The FACEs method extracts informative features from longitudinal data and presents them as scores. It reveals good performance in the case of sparse longitudinal data like the current study.

Next, the features extracted were included in the random survival forest model (RSF) as time-independent covariates along with the two baseline variables. RSF is an extension of the random forest approach, a non-parametric machine learning algorism, to survival analysis [18]. The model is constructed by averaging the predicted hazards of many decision trees. Unlike conventional survival methods, RSF bypasses the assumption of proportional hazards and provides a way to handle unspecified interactions [22] and patterns of non-linearity [23] in the covariates. RSF in the current study was built using 1000 trees and other default parameters.

We developed a baseline model using RSF based on the same predictor variables as in the longitudinal model, but utilizing only a single measurement taken at baseline. Additionally, for ease of interpretation, we constructed another baseline model using Cox regression.

### Statistical analysis

Continuous variables were summarized as mean (standard deviation), and categorical variables were summarized as count (percentage). The follow-up time of patients was presented as median (range). Continuous variables were compared using the Student t-test or the Mann-Whitney U test, and categorical variables by either the Chi-square test or the Fisher exact test when appropriate.

The averaged trajectory of each longitudinal predictor was estimated using mixed-effects models with random and fixed effects for measurement time. In addition to a linear model, a non-linear model which included natural cubic splines with 2 degrees of freedom in both the random and fixed effects part was also constructed.

The study population was split into a training set (70%) and a validation set (30%) at random. The baseline and longitudinal models were developed on the training set and assessed on the validation set. We presented the performance of the two models in predicting HCC occurrence in subsequent 1, 2, and 3 years after the third year of follow-up. In essence, the longitudinal information in the initial 3 years of follow-up served as the basis for the prediction of events happening in the 4th, 5th, and 6th years of follow-up. We chose three years from enrollment as the prediction window because the time frame allowed for the majority of patients in the validation set (81.25%) to have at least two repeated measurements recorded.

To further evaluate the robustness of our modeling strategy, we employed a leave-one-out cross-validation approach. For each iteration, we trained the model using the entire dataset, excluding one patient’s data, and then tested the model on the omitted patient. This process was repeated for every individual, resulting in a predicted probability of HCC occurrence for each patient. The prediction window was still set at three years from enrollment.

Across both our main model and the leave-one-out validation, the predictive performance of the models was evaluated in terms of both discrimination and calibration. The time-dependent areas under the receiver-operating characteristics curves (AUROC) were used to measure the discriminatory capacity of the models for separating patients who developed HCC 1, 2, and 3 years after Year 3 from patients who did not [24, 25]. A higher AUROC indicates better model performance. Brier scores, which capture both discrimination and calibration, were used as a metric for overall accuracy. Brier scores range between 0 and 1, with scores closer to 0 representing higher accuracy and better model performance. To adjust for right censoring, the Kaplan–Meier method was used as the inverse probability of censoring weights estimator in calculating brier scores [24]. The 95% confidence interval (CI) of the time-dependent AUROC and brier score were estimated based on 1000 bootstrap samples of the validation set.

Statistical significance was set at P < 0.05. Data analysis was all performed using R software, version 4.1.2 (R Foundation for Statistical Computing) [26]. The FACEs method was performed using the face package [27], and the RSF was constructed using the randomForestSRC package [28]. The risk prediction of RSF models was performed with the pec package [29]. The time-dependent AUROC was calculated using the tdROC package [30]. The online web calculator was constructed using the shiny package [31].

## Results

### Baseline characteristics of patients

A total of 400 HCV-infected patients with cirrhosis were enrolled in the study. 280 patients (70%) were randomized to the training set and 120 (30%) to the validation set. The characteristics of patients in the two groups are displayed in Table 1. There were no significant differences in baseline characteristics between these two groups. Both groups consisted mainly of female patients (72.5-77.9%) around 60 years old (60.21 and 60.43 years). During a median follow-up of approximately 5 years (4.76 and 4.84 years), 25 patients (8.9%) in the training set and 11 patients (9.2%) in the validation set developed HCC. Patients with the longest follow-up time were followed for 10.96 years in the training set and 10.87 years in the validation set. The Kaplan-Meier curve of the cumulative probability of HCC in the entire study population is shown in Figure S2.

**Table 1:** Baseline Characteristics of Patients*

Characteristics	Training set (n = 280)	Validation set (n = 120)	*P* value
Age (years)	60.21 (7.10)	60.43 (6.71)	0.767
Gender, female	218 (77.9)	87 (72.5)	0.305
AFP (ng/ml)	13.21 (25.37)	11.30 (21.79)	0.472
Total Bilirubin (µmol/L)	20.62 (13.98)	21.87 (26.82)	0.542
Direct Bilirubin (µmol/L)	7.24 (7.82)	8.01 (16.67)	0.530
ALT (U/L)	82.97 (111.41)	73.40 (62.72)	0.378
AST (U/L)	72.37 (65.65)	68.70 (55.95)	0.593
Cholinesterase (U/L)	5917.30 (1921.28)	6137.38 (1738.98)	0.281
ALP (U/L)	93.00 (34.42)	87.58 (36.87)	0.158
GGT (U/L)	70.71 (67.31)	64.12 (72.92)	0.382
Total Protein (g/L)	79.38 (8.47)	78.76 (6.35)	0.471
Albumin (g/L)	43.65 (5.49)	44.67 (6.45)	0.106
Median follow-up time (years) [range]	4.76 [0.51,10.96]	4.84 [0.32,10.87]	0.083
HCC	25 (8.9)	11 (9.2)	1.000

* Continuous variables were presented as mean (standard deviation), and categorical variables were presented as count (percentage)

AFP, alpha-fetoprotein; ALT, alanine aminotransferase; AST, aspartate aminotransferase; ALP, alkaline phosphatase; GGT, gamma-glutamyl transferase; HCC, hepatocellular carcinoma

### Trajectories of longitudinal predictors over time

**Fig. 1:**
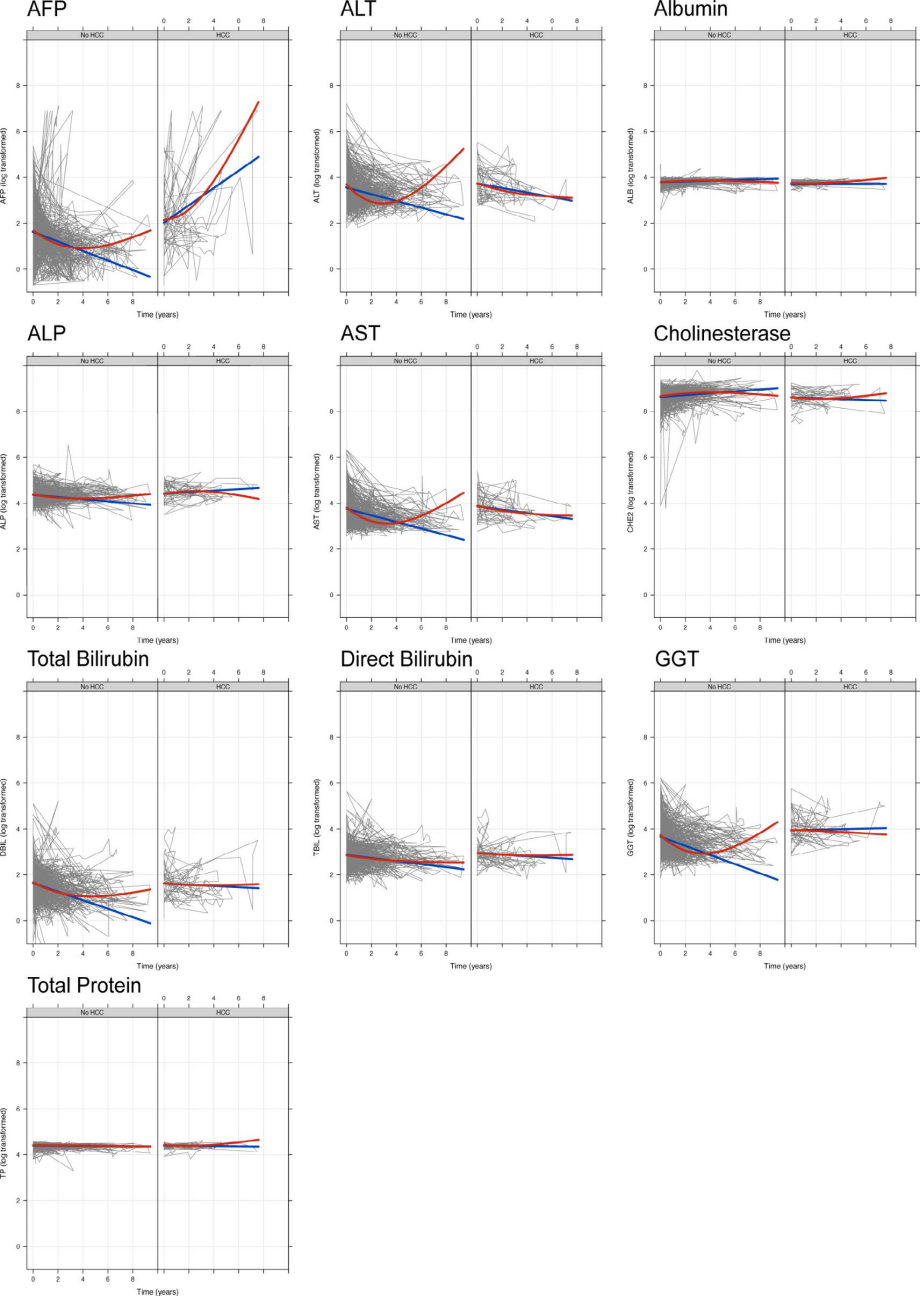
Trajectories of 10 longitudinal predictors in patients with HCC and without from the entire cohort. The longitudinal predictors were AFP (a), ALT (b), albumin (c), ALP (d), AST (e), cholinesterase (f), direct bilirubin(g), GGT (h), total bilirubin (i), and total protein (j). The grey lines represent individual trajectories of each patient, the blue lines are the averaged trajectories estimated using linear mixed-effects models and the red lines are the averaged trajectories estimated using mixed-effects models that includes natural cubic splines with 2 degrees of freedom. The values of all predictor variables are on a log scale. Abbreviations: AFP, alpha-fetoprotein; ALT, alanine aminotransferase; AST, aspartate aminotransferase; ALP, alkaline phosphatase; GGT, gamma-glutamyl transferase; HCC, hepatocellular carcinoma

The patients’ longitudinal predictors have been measured an average of 3.68 times (range 1–26) during the entire follow-up period. To illustrate the evolution of these predictors over time, we presented in Fig. 1 the individual and averaged trajectories of the 10 longitudinal predictors in patients who developed HCC and those who did not from the entire cohort.

As can be seen, the trajectory of the longitudinal predictors differed in patients who had HCC and patients who did not. For example, the AFP level in patients who experienced HCC increased dramatically during the follow-up period, while the AFP level in patients who did not experience HCC remained stable or even decreased steadily. Also, the GGT level in patients who did not develop HCC appeared to decrease over time, whereas the GGT level in patients who developed HCC remained stable.

### Individual-level prediction of HCC-free probabilities

**Fig. 2:**
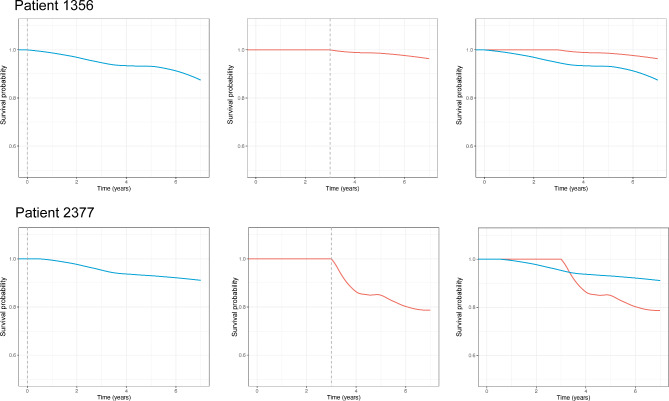
Individual-level prediction of HCC-free probabilities for two patients from the validation set. Survival curves were smoothed with local polynomial regression. The blue lines represent the HCC-free probabilities predicted by the baseline RSF model, and the orange lines represent the HCC-free probabilities predicted by the longitudinal model. Survival curves were overlaid in the final column. Abbreviations: HCC, hepatocellular carcinoma; RSF, random survival forest

Figure 2 illustrates the predictions made with the longitudinal models and the baseline RSF model for two patients with similar biomarker values at baseline from the validation set. The predictions of the longitudinal model were made at Year 3 of the follow-up. It can be noted that the baseline model gave similar predictions for HCC risk in the two patients. In contrast, the longitudinal model indicated a sharp increase in HCC risk for patient 2377 and a relatively low risk for patient 1356. As demonstrated by the overlaid survival curves, the longitudinal model assigned a higher survival probability to patient 1356 and a lower survival probability to patient 2377 compared with the baseline model. The predictions made by the longitudinal model were consistent with the actual outcome, with patient 2377 developing HCC 4.85 years after enrollment. In contrast, patient 1356 had not developed HCC at the time of last clinical visit, 5.65 years after enrollment.

### Performance of prediction models in the validation set

**Fig. 3:**
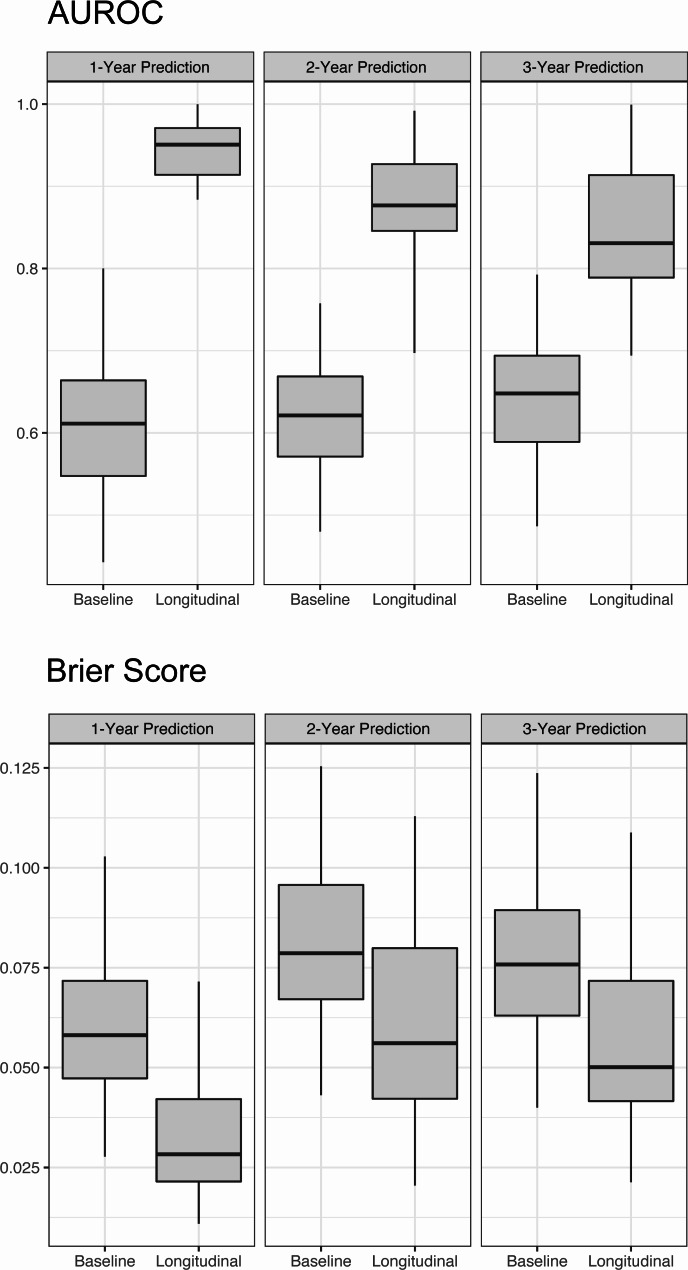
Area under the receiver operating characteristic curves value and brier score of the baseline RSF model and longitudinal model for predictions made 1, 2, and 3 years from Year 3. Predictions were made at Year 3 for HCC occurrence 1, 2, and 3 years from Year 3, which equals 4, 5, and 6 years from baseline. Abbreviations: HCC, hepatocellular carcinoma; AUROC, area under the receiver-operating characteristic curve; RSF, random survival forest

Validation of the models was performed on a random 30% split of the entire study cohort. The validation set was not included in model development. Three years after enrollment, 94 out of 120 patients in the validation set were still at risk of HCC. In this subset of patients, the longitudinal model showed excellent performance in predicting HCC events that occurred 1 year after, with an AUROC of 0.9507 (95% CI 0.8838–0.9997). For 2-year and 3-year predictions, the performance of the longitudinal model was very good as well, with AUROCs both above 0.8 (0.8767 and 0.8307, respectively). Additionally, the longitudinal model achieved remarkably low brier scores in the 1-, 2- and 3- year predictions of HCC (0.0283, 0.0561, and 0.0501, respectively). In comparison, the baseline model also constructed with RSF only achieved mediocre AUROCs in predicting HCC events 1, 2, and 3 years from Year 3 (0.6113, 0.6213, and 0.6480, respectively) (Table 2). As is demonstrated in Fig. 3, the longitudinal model outperformed the baseline RSF model with better discriminative accuracy and improved calibration. The Cox regression baseline model showed performance similar to the RSF baseline model (Table S1).

**Table 2:** Comparison of the Performance Characteristics of the Longitudinal and Baseline RSF Models to Predict the Development of HCC*

	Longitudinal (95% CI)	Baseline Only (95% CI)
1-Year Prediction		
AUROC	0.9507 (0.8838,0.9997)	0.6113 (0.4428,0.8000)
Brier score	0.0283 (0.0109,0.0715)	0.0581 (0.0277,0.1028)
2-Year Prediction		
AUROC	0.8767 (0.6972,0.9918)	0.6213 (0.4801,0.7575)
Brier score	0.0561 (0.0205,0.1129)	0.0786 (0.0431,0.1254)
3-Year Prediction		
AUROC	0.8307 (0.6941,0.9993)	0.6480 (0.4865,0.7924)
Brier score	0.0501 (0.0213,0.1088)	0.0758 (0.0400,0.1237)

*Predictions were made at Year 3 for HCC occurrence 1, 2, and 3 years from Year 3, which equals 4, 5, and 6 years from baseline

HCC, hepatocellular carcinoma; CI, confidence interval; AUROC, area under the receiver-operating characteristic curve; RSF, random survival forest

The relative importance of the 10 predictors that contributed the most to the performance of the longitudinal model was displayed in Figure S3. The longitudinal predictor AFP contributed highly to the prediction of HCC with large variable importance (VIMP) compared to other predictors. Other key longitudinal predictors identified by VIMP include GGT, direct bilirubin, total bilirubin, albumin, and ALP. Also, age at baseline was very informative in predicting HCC development.

Given the significant contribution of AFP to the longitudinal model’s performance, we explored a model solely based on AFP levels (Table S2). This ‘AFP-only’ model demonstrated promising results, particularly for predicting HCC within the subsequent year, achieving an AUROC of 0.8297. However, its efficacy waned over extended periods, with the AUROC of 3-year prediction dropping to 0.6383.

### Evaluation of prediction model performance using leave-one-out cross-validation

The efficacy of our longitudinal modeling approach was further assessed using leave-one-out cross-validation. The longitudinal model fitted with the entire dataset demonstrated commendable predictive capabilities, as detailed in Table S3. The AUROC in predicting HCC events 1, 2, and 3 years from Year 3 was 0.8504, 0.7235, and 0.7173, respectively. Notably, the longitudinal model consistently outperformed the baseline RSF model in our evaluations.

## Discussion

The DAA-based regimen has brought revolutionary changes to the management of chronic hepatitis C as it offers excellent rates of HCV virus eradication [3, 4]. With the widespread use of DAA, it is foreseeable that most HCV-infected patients will achieve SVR. However, multiple studies have shown that the residual risk of HCC persists years after patients achieved SVR [32, 33] and that patients with cirrhosis present a significantly higher risk of HCC post-SVR than patients with no cirrhosis [5, 34]. Currently, only less than 50% of patients with cirrhosis undergo regular surveillance in most healthcare systems [35]. As a result, it is critical to determine which patients with cirrhosis need HCC surveillance the most, especially in healthcare systems with limited resources. Risk prediction models can provide valuable insights in guiding clinical decisions. Most risk prediction models for HCC occurrence were constructed on data collected at baseline alone and thus cannot capture the changes in the predictor variables, resulting in a loss of information. In this study, we aimed to construct an accurate longitudinal prediction model for HCC occurrence based on repeated measurement data. We also demonstrated the predictive accuracy of the longitudinal model through comparisons with a baseline model.

400 patients with HCV-related cirrhosis were included in the study, with a median follow-up time of approximately 5 years. The patient with the longest follow-up period was followed for up to 10.96 years. The longitudinal predictors were measured on average 3.68 times. We modeled the average trajectory of the longitudinal predictors and found that the trajectories of the predictors differ in patients with or without HCC, indicating the necessity of developing a longitudinal prediction model.

The current study employed a two-step modeling framework to capture the changes in the predictor variables and apply them in the predictive model. FACEs, a covariance-based FPCA method, was used to extract informative features from the trajectories of the longitudinal predictors. The resulting PCA scores were then included as time-independent covariates in the RSF. Our model yielded predictive accuracy that is considered excellent [36] in the validation set for 1-year prediction, with an AUROC of 0.9507. For 2- and 3-year prediction, the model also exhibited very good predictive accuracy, with AUROCs of 0.8767 and 0.8307, respectively. Equally importantly, our model had excellent calibration, as demonstrated by the very low brier scores for 1-,2- and 3- year predictions.

In addition, we constructed a baseline model with RSF using the same predictor variables. Our results showed that the longitudinal model outperformed the baseline RSF model at individual and population levels. For two patients with similar baseline measurements, the longitudinal model made accurate personal predictions that corresponded with their distinct outcomes, whereas the baseline model failed to distinguish between the HCC risk of the two patients. Also, the baseline model returned a mediocre AUROC of around 0.6 in predicting HCC occurrence 1-,2- and 3- years after Year 3 of the follow-up. In contrast to the longitudinal model, the predictive accuracy of the baseline model was only considered sufficient [36].

In clinical practice, obtaining all the predictive variables used in the model can be challenging. To address this, we constructed an alternative model exclusively based on repeated AFP measurements. Prior research has already highlighted the efficacy of AFP levels in predicting HCC in patients with chronic liver disease [36]. Our ‘AFP-only’ model also displayed satisfactory results, particularly for one-year predictions. Although the prediction performance of the simplified model diminished for long-time prediction, this model still offers a practical solution when data for all variables are not available, especially for making short-term predictions.

To enhance the clinical applicability of our longitudinal model, we have developed a web application to assess the risk of HCC development in patients. This online tool offers two options: the comprehensive model, as proposed in this study, and a simplified ‘AFP-only’ model that leverages only the longitudinal AFP data. Clinicians can select the appropriate model based on their available data. A preliminary version of this tool is available at https://shizongrenkou.shinyapps.io/HCC_calculator/. For users who prefer a local solution, all codes and dependencies can be found on our GitHub repository (https://github.com/shizongrenkou/HCC.cal). Once downloaded, the app can be executed locally in R, providing a faster user experience without the constraints of the online version.

Our study holds the following strengths. First, the study utilized a novel statistical approach to construct a predictive model incorporating longitudinal data. Most risk prediction models for HCC development were built using the Cox regression [11, 37] or logistic regression [14]. These conventional regression modeling algorithms cannot capture the changes in predictor variables during follow-up; hence the model-building process relies entirely on baseline data. A few studies expanded the data collection window to baseline and a single follow-up time point [38, 39]. This modeling strategy is still rather rigid and discards potentially valuable information from the longitudinal data. There have been studies that utilize longitudinal information for risk prediction in hepatitis C patients. A study that aimed to construct prediction models for liver-related outcomes employed a joint modeling framework to incorporate longitudinal data into the modeling process [40]. Though the longitudinal model performed very well in predicting incidences of decompensation (AUROC = 0.92), it did poorly in predicting the occurrence of HCC (AUROC = 0.59). In contrast, the longitudinal model in the current study yielded excellent predictive accuracy of HCC incidence.

Furthermore, the current study adopted the RSF method to analyze the right-censored survival data. The RSF is an extension of the random forest algorithm. While random forest focuses mainly on classification and regression, RSF further accounts for right-censoring and extends the application of machine learning to survival analysis [18]. The majority of the studies employing machine learning methods in HCC risk prediction are inclined to use the classification-based method like the random forest or boosting [41, 42]. There was also a study that leveraged deep learning methods to predict whether a specific patient with cirrhosis would develop HCC within the next 3 years using all longitudinal data available at the prediction time point [43]. The deep learning model exhibited an AUROC of 0.759, which is considered very good. However, the modeling strategy of the study was still classification-based. These studies are limited when dealing with survival data as the machine learning classifiers cannot predict the time to an outcome, do not account for censoring, and needs to be re-trained for each prediction time [44]. The RSF, on the other hand, addresses these issues effectively. The RSF also holds advantages over the commonly used Cox regression in handling survival data. It avoids the restrictive assumption of proportional hazards, tackles the non-linear effects of variables, and automatically handles the interactions between multiple variables [22, 29]. This flexibility allows RSF to have more robust discrimination and calibration in risk prediction.

Lastly, our longitudinal model was trained on data with irregular time intervals and still performed well in validation. In some longitudinal studies, the repeated measurements of patients were done on fixed time points, like annually or semi-annually [41]. However, in real-world clinical practice, patients often come to follow-up visits at random times. Therefore, these longitudinal models cannot be applied to such situations. Our model was trained on longitudinal data with irregular time intervals and is better suited for clinical use.

There are several limitations to our study. First, the current study employed the fast covariance estimation method (FACEs) instead of the multivariate fast covariance estimation method (mFACEs) [45] suggested by the original article in dealing with longitudinal data due to computational cost. As a result, the correlation between the longitudinal variables was omitted when extracting the informative features. Nevertheless, the model performed well in terms of predictive accuracy. Second, the study’s validation set was relatively small, which might explain the large confidence interval of AUROCs for 2-and 3-year prediction of the longitudinal model. Third, some of the patients did not come back for follow-up visits, so only baseline measurements were available for these patients. Fourth, to ensure sufficient repeated measurements, we selected a three-year window from enrollment as the prediction timeframe. While this decision was made to accumulate a more robust set of longitudinal information, it may have introduced a selection bias. Finally, the study cohort was all Asian and consisted mainly of females. It is necessary to perform external validations of our model in other cohorts.

In conclusion, the current study demonstrated that predictive model constructed on longitudinal data performed better than baseline model in estimating the risk of HCC occurrence in patients with HCV-related cirrhosis. Our longitudinal model performed especially well in predicting the occurrence of HCC within one year. Our model could have a variety of applications in clinical practice. The model is particularly useful in resource-limited countries that do not have the capacity to offer surveillance to all cirrhotic patients, as it identifies high-risk patients based on a few simple laboratory biomarkers. Our model could also be used to identify high-risk patients for novel and relatively expensive surveillance strategies. Further studies and a larger population will be needed to validate our results externally.

### Electronic supplementary material

Below is the link to the electronic supplementary material.

Supplementary Material 1

## Data Availability

The datasets analyzed during the current study are not publicly available due to the privacy of individuals who participated in the study but are available from the corresponding author upon reasonable request.

## List of abbreviations

HCC: hepatocellular carcinoma
SVR: sustained virological response
DAA: direct-acting antivirals
HCV: hepatitis C virus
RSF: random survival forest
AUROC: area under the receiver-operating characteristics curve
AFP: alpha-fetoprotein
AST: aspartate aminotransferase
ALT: alanine transaminase
GGT: gamma-glutamyl transferase
ALP: alkaline phosphatase
FACEs: fast covariance estimation method
FPCA: functional principal component analysis
CI: confidence interval
VIMP: variable importance
