# Supplementary material for: Accurate prediction of HCC risk after SVR in patients with hepatitis C cirrhosis based on longitudinal data
Source: BMC Cancer. 2023 Nov 25;23:1147. doi: 10.1186/s12885-023-11628-1 (PMC10676612; doi:10.1186/s12885-023-11628-1)
Supplement: Supplementary file 1 — Supplementary Material 1 [file 12885_2023_11628_MOESM1_ESM.docx]

**Supplement figure 1:** Flow diagram of patient selection.

Abbreviations: DAA, direct-acting antivirals; SVR, sustained virologic response; HCC, hepatocellular carcinoma.

**Supplement figure 2:** Kaplan-Meier curve of the cumulative probability of HCC in the entire study population.

Abbreviations: HCC, hepatocellular carcinoma.

**Supplement figure 3:** Variable importance of the 10 key predictors in the longitudinal model.

Abbreviations: AFP, alpha-fetoprotein; ALP, alkaline phosphatase; GGT, gamma-glutamyl transferase; PC1, first principal component score; PC2, second principal component score;

VIMP, variable importance.

**Supplement table 1:** Performance characteristics of the baseline model constructed with Cox regression to predict the development of HCC*

|  | **Baseline Cox (95%CI）** |
| --- | --- |
| 1-Year Prediction |  |
| AUROC | 0.6208 (0.4309,0.8985) |
| Brier score | 0.0678 (0.0283,0.1143) |
| 2-Year Prediction |  |
| AUROC | 0.5898 (0.3782,0.8140) |
| Brier score | 0.1134 (0.0525,0.1801) |
| 3-Year Prediction |  |
| AUROC | 0.5978 (0.3806,0.8299) |
| Brier score | 0.1155 (0.0575,0.1800) |

*Predictions were still made at Year 3 for HCC occurrence 1, 2, and 3 years from Year 3, which equals 4, 5, and 6 years from baseline, the same as the longitudinal model and the baseline RSF model.

Abbreviations: HCC, hepatocellular carcinoma; CI, confidence interval; AUROC, area under the receiver-operating characteristic curve; RSF, random survival forest.

**Supplement table2:** Performance characteristics of the longitudinal and baseline models that only incorporate AFP (the ‘just AFP’ model) in predicting the development of HCC*

|  | **Longitudinal (95% CI)** | **Baseline Only (95% CI)** |
| --- | --- | --- |
| 1-Year Prediction |  |  |
| AUROC | 0.8297 (0.4727,0.9993) | 0.5844 (0.5335,0.6514) |
| Brier score | 0.0260 (0.0122,0.0731) | 0.0593 (0.0465,0.0735) |
| 2-Year Prediction |  |  |
| AUROC | 0.7449 (0.4558,0.9892) | 0.4931 (0.4338,0.5532) |
| Brier score | 0.0498 (0.0225,0.1166) | 0.0784 (0.0633,0.0936) |
| 3-Year Prediction |  |  |
| AUROC | 0.6383 (0.4498,0.9869) | 0.4266 (0.3723,0.4858) |
| Brier score | 0.0541 (0.0238,0.1101) | 0.0820 (0.0669,0.0956) |

*Predictions were made at Year 3 for HCC occurrence 1, 2, and 3 years from Year 3, which equals 4, 5, and 6 years from baseline.

Abbreviations: HCC, hepatocellular carcinoma; CI, confidence interval; AUROC, area under the receiver-operating characteristic curve.

**Supplement table3:** Performance characteristics of the longitudinal and baseline models when evaluated with a leave-one-out cross-validation approach*

|  | **Longitudinal** | **Baseline Only** |
| --- | --- | --- |
| 1-Year Prediction |  |  |
| AUROC | 0.8504 | 0.5737 |
| Brier score | 0.0180 | 0.0598 |
| 2-Year Prediction |  |  |
| AUROC | 0.7235 | 0.5574 |
| Brier score | 0.0289 | 0.0665 |
| 3-Year Prediction |  |  |
| AUROC | 0.7173 | 0.5308 |
| Brier score | 0.0331 | 0.0687 |

*Predictions were made at Year 3 for HCC occurrence 1, 2, and 3 years from Year 3, which equals 4, 5, and 6 years from baseline. Three years after enrollment, 315 out of 400 patients in the entire dataset were still at risk of HCC. The validation of the longitudinal model was based on this group of patients.

Abbreviations: HCC, hepatocellular carcinoma; CI, confidence interval; AUROC, area under the receiver-operating characteristic curve.
